# Professor Virchow

**Published:** 1860-01

**Authors:** 


PROFESSOR VIRCHOW.—The vacancy occasioned in the French Academy of Sciences, by the death of Dr. Marshall Hall, has been filled by the appointment of the celebrated Berlin professor.

